# Local Applications to the Upper Air Passages

**Published:** 1907-11-23

**Authors:** 


### LARYNGOLOGY AND RHINOLOGY.

### LOCAL APPLICATIONS TO THE UPPER AIR PASSAGES.

THE prescription of medicaments for local application to the nose and throat, and the best methods of using them, are often somewhat vaguely described in the text-books; remedies not infrequently fail of their effect because they are not properly applied, for lack of complete directions as to the correct method of use. It is extraordinary in how many ways some patients will go wrong in the manipulation of such a simple instrument as the nasal syringe. The most concise instructions are necessary, and the patient should be taught the proper method under the eye of the physician.

### NASAL LOTIONS.

Nasal lotions, or collunaria, are a most valuable means of treating inflammatory conditions of the nose, naso-pharynx, or fauces, and are also of use in laryngitis arising by extension from these regions. As is well known, gargles do not as a rule penetrate beyond the anterior faucial pillars, whereas a lotion introduced through the nose will reach the fauces far more effectively. The nasal mucous membrane is very delicate, and strong antiseptics and astringents must never be used in the lotion; plain water also should be avoided, as it injures the cells by osmosis. Thus normal saline solution, a teaspoonful of table-salt to the pint of warm water, answers very well where a simple mechanical action is required; as a rule a mild alkaline lotion is preferable by reason of its solvent action on the mucus; two or three grains of sodium bicarbonate to the ounce does very well. A mild antiseptic is generally added, such as borax 3 grains, and carbolic acid 1 grain to the ounce. To give "body" to the solution, so that it remains longer in contact with the parts, 5 grains of sugar or 45 minims of glycerine should be employed. Thus we get a simple lotion such as the following: Sodium bicarbonate gr. 3, sodium biborate gr. 3, carbolic acid gr. 1, glycerine ♏45, water to 1 oz. But these lotions must always be used warm, at a temperature of about 90° F.; therefore it is better to prescribe the lotion thrice as strong and to order its dilution with two parts of warm water; or it may conveniently be given in the form of a powder for solution: Sodium bicarbonate, sodium biborate, and potassium chlorate, of each one part, white sugar two parts; a teaspoonful to be dissolved in a tumblerful of warm water. As mentioned above, strong antiseptics or astringents should not be used. Neither do I advise the addition of cocaine or adrenalin to a nasal lotion, for the vaso-constrictor effect is transient, and is followed by dilatation. Hazeline may be used in the proportion of 20 minims to the ounce of the diluted lotion; of antiseptics listerine in the same strength, or Sanitas 30 drops to the ounce, may be recommended for ozænic or suppurative cases.

The method of application is important. The lotion may be sniffed up from the hand, a saucer, or a spoon, and returned through the mouth; but many patients fail to do this properly, and at best it is not a very efficient method. Small glass douches are sold from which the lotion may be poured into the nose when the head is tilted backwards; the fluid passes with a gush into the pharynx and often down the larynx, and there is a distinct danger that it may enter the eustachian tube. A syphon douche should never be employed, for the pressure is apt to be too great, and is continuous: the lotion may then readily reach the middle ear. A small rubber ball-syringe is the best instrument for most cases, as it is the most comfortable to use and the least dangerous. The patient should be carefully instructed to use it in the following way: Leaning slightly forward over a basin, he fits the syringe into one nostril, so as to direct the stream backwards along the floor of the nose; he then empties the syringe slowly, breathing deeply the while through the open mouth; if the nose be fairly patent the lotion will return by the other nostril, for the palate is raised and shuts off the lower part of the pharynx. He syringes through each nostril alternately until he has used 8 or 10 oz. of the lotion. To obviate the risk of forcing fluid into the eustachian tube, certain precautions should be insisted on. Thus he must use no force, and should apply the syringe to the most obstructed nostril first, and, if there be permanent obstruction of one side, as from a deflected septum, he should syringe only through that side. Further, he should not blow the nose in the ordinary way, which occludes both nostrils, for at least fifteen minutes after syringing, but he may close one nostril and blow down the other, provided it be fairly patent.

These precautions may appear elaborate to fussiness, but acute otitis media with all its complications has occurred many times as the result of careless syringing of the nose. The eustachian tube is especially patent in children, and it is certainly safer not to order syringing as a routine measure after the removal of adenoids; the wound heals quite well without local treatment. After operations on the nose, such as removal of parts of the turbinals, it is better to use a syringe with a fine nozzle which does not block the nostril so that it is impossible to pump the fluid in under pressure; and in cases of disease of the maxillary antrum or frontal sinus a suitably curved nozzle should be provided after the operation, and the patient taught to pass it correctly into the cavity. In the case of infants and very young children syringing is difficult, and the danger to the ears is still greater; it is better to drop the lotion into the nose, as the child lies on the back, from a spoon or from the filler of a stylographic pen.

If the syringe be used as above directed, it will rarely cause discomfort, but some patients complain of heaviness or headache. In such cases the lotion may be used as a spray, though this is less effective than the syringe as a cleansing application. A coarse spray should be chosen, when it is intended as a substitute for the syringe, to act on the nasal mucous membrane, and is not meant to reach the larynx or trachea; fine sprays, or nebulæ, for applying medicaments to the larynx, etc., will be considered in a subsequent article.

*(To be continued.)*

